# Verifying Three-Dimensional Skull Model Reconstruction Using Cranial Index of Symmetry

**DOI:** 10.1371/journal.pone.0074267

**Published:** 2013-10-25

**Authors:** Woon-Man Kung, Shuo-Tsung Chen, Chung-Hsiang Lin, Yu-Mei Lu, Tzu-Hsuan Chen, Muh-Shi Lin

**Affiliations:** 1 Department of Exercise and Health Promotion, College of Education, Chinese Culture University, Taipei, Taiwan; 2 Department of Neurosurgery, Lo-Hsu Foundation, Lotung Poh-Ai Hospital, Luodong, Yilan, Taiwan; 3 Department of Applied Mathematics, Tunghai University, Taichung, Taiwan; 4 Department of Computer Science and Information Engineering, College of Electrical Engineering and Computer Science, National Taiwan University, Taipei, Taiwan; 5 Department of Health Promotion and Health Education, National Taiwan Normal University, Taipei, Taiwan; 6 Department of Physical Medicine and Rehabilitation, Mackay Memorial Hospital, Hsinchu, Taiwan; 7 Department of Surgery, Faculty of Medicine, School of Medicine, National Yang-Ming University, Taipei, Taiwan; 8 Department of Neurosurgery, Taipei City Hospital, Zhong Xiao Branch, Taipei, Taiwan; 9 Institute of Biomedical Engineering, College of Medicine and College of Engineering, National Taiwan University, Taipei, Taiwan; Johns Hopkins Hospital, United States of America

## Abstract

**Background:**

Difficulty exists in scalp adaptation for cranioplasty with customized computer-assisted design/manufacturing (CAD/CAM) implant in situations of excessive wound tension and sub-cranioplasty dead space. To solve this clinical problem, the CAD/CAM technique should include algorithms to reconstruct a depressed contour to cover the skull defect. Satisfactory CAM-derived alloplastic implants are based on highly accurate three-dimensional (3-D) CAD modeling. Thus, it is quite important to establish a symmetrically regular CAD/CAM reconstruction prior to depressing the contour. The purpose of this study is to verify the aesthetic outcomes of CAD models with regular contours using cranial index of symmetry (CIS).

**Materials and methods:**

From January 2011 to June 2012, decompressive craniectomy (DC) was performed for 15 consecutive patients in our institute. 3-D CAD models of skull defects were reconstructed using commercial software. These models were checked in terms of symmetry by CIS scores.

**Results:**

CIS scores of CAD reconstructions were 99.24±0.004% (range 98.47–99.84). CIS scores of these CAD models were statistically significantly greater than 95%, identical to 99.5%, but lower than 99.6% (p<0.001, p = 0.064, p = 0.021 respectively, Wilcoxon matched pairs signed rank test). These data evidenced the highly accurate symmetry of these CAD models with regular contours.

**Conclusions:**

CIS calculation is beneficial to assess aesthetic outcomes of CAD-reconstructed skulls in terms of cranial symmetry. This enables further accurate CAD models and CAM cranial implants with depressed contours, which are essential in patients with difficult scalp adaptation.

## Introduction

Under critical circumstances, such as severe traumatic brain injury (TBI) and massive stroke, a wide decompressive craniectomy (DC) reduces increased intracranial pressure (IICP) and consequently might improve the long-term outcome [1]–[3]. In surviving patients, however, functional and aesthetic reconstruction of skull defect following DC is imperative, especially in young patients, who concern much about their appearances [4].

The ideal material for cranioplasty is autogenous bone. In many cases, the original bone flap is not used because there is not enough available due to fracture, or not in the proper shape. Furthermore, bones with osteolysis or infection may render them unusable [4], [5]. In comparison, there is much advancement in the development of computer-assisted design/manufacturing (CAD/CAM) - fabricated implants in the repair of skull defects after DCs. A growing body of literature addresses the advantages using three-dimensional (3-D) CAD implants for craniofacial skeletal reconstruction in terms of excellent cosmesis and few complications when compared to conventional autogenous or alloplastic bone grafts in the repair of large skull defects [5]–[8].

In patients following DCs, difficulties may exist in scalp adaptation during subsequent cranioplastic surgeries due to scalp contraction, scalp atrophy, paucity of soft tissue [9] secondary to wound infection, poor nutrition, and tense sutures [10]. In such situations, the scalp tissue is insufficient to cover the reconstructed skull. Furthermore, excessive scalp wound tension may result in wound breakdown, scar alopecia and poor wound healing [11]. Therefore, using a smaller implant for alloplastic cranioplasty to minimize stretching of the scalp is obviously essential. In clinical practice, the CAD/CAM technique involves algorithms to reconstruct depressing contours to prevent excessive tension to the scalp. However, creating CAM-derived alloplastic implants with reduced height may lose cosmetic outcomes in terms of symmetry. The optimal height for depression in CAD/CAM implant designs has been an area of significant research interest in skull defect reconstruction.

Prior to depressing the contour of 3-D computer tomography (CT) reconstructions, it is quite important to establish a verified regular model without depressed contours. In the present study, we sought to apply the cranial index of symmetry (CIS) method to access the symmetry of regular 3-D reconstruction of skull skeletons. The overall objective of this study is to provide a novel inspection protocol to verify regular CAD reconstructions in terms of symmetry.

## Materials and Methods

### Ethics statement

All patients provided their written informed consent and all procedures were approved by the ethics committees of Taipei City Hospital in accordance with Declaration of Helsinki.

### Patients

From January 2011 to June 2012, 15 consecutive patients aged 34–90 years with intracranial hematomas due to TBI and massive stroke were treated in our institute. All 15 patients presented with marked neurological deficits and underwent cranial decompressive surgery. The operative method included a wide DC with the removal of a hematoma on the lesioned side (n = 15). We reviewed the CT scans of these patients with skull defects.

Patients received CT scans of the brain with slice thickness of 1.25 mm from the skull base to convexity, including the region of the skull defect [4]. The thin-sliced high-resolution CT images were prepared for the 3-D reconstruction of skull skeletons.

### 3-D CAD reconstruction of skull defects

Technical advancements in hardware and software make it feasible to create accurate 3-D CAD reconstructions of skull skeletons [12]–[14]. A wide variety of commercial software is useful to reconstruct skull CAD models, such as Open Source Computer Vision (OpenCV), Open Graphic Library (OpenGL) OBJ Viewer, GLC Player, etc. Herein, we demonstrate our CAD algorithm using OpenCV and OpenGL OBJ Viewer.

### Editing the 2-D images of brain CT scans using OpenCV

**Figure 1:**
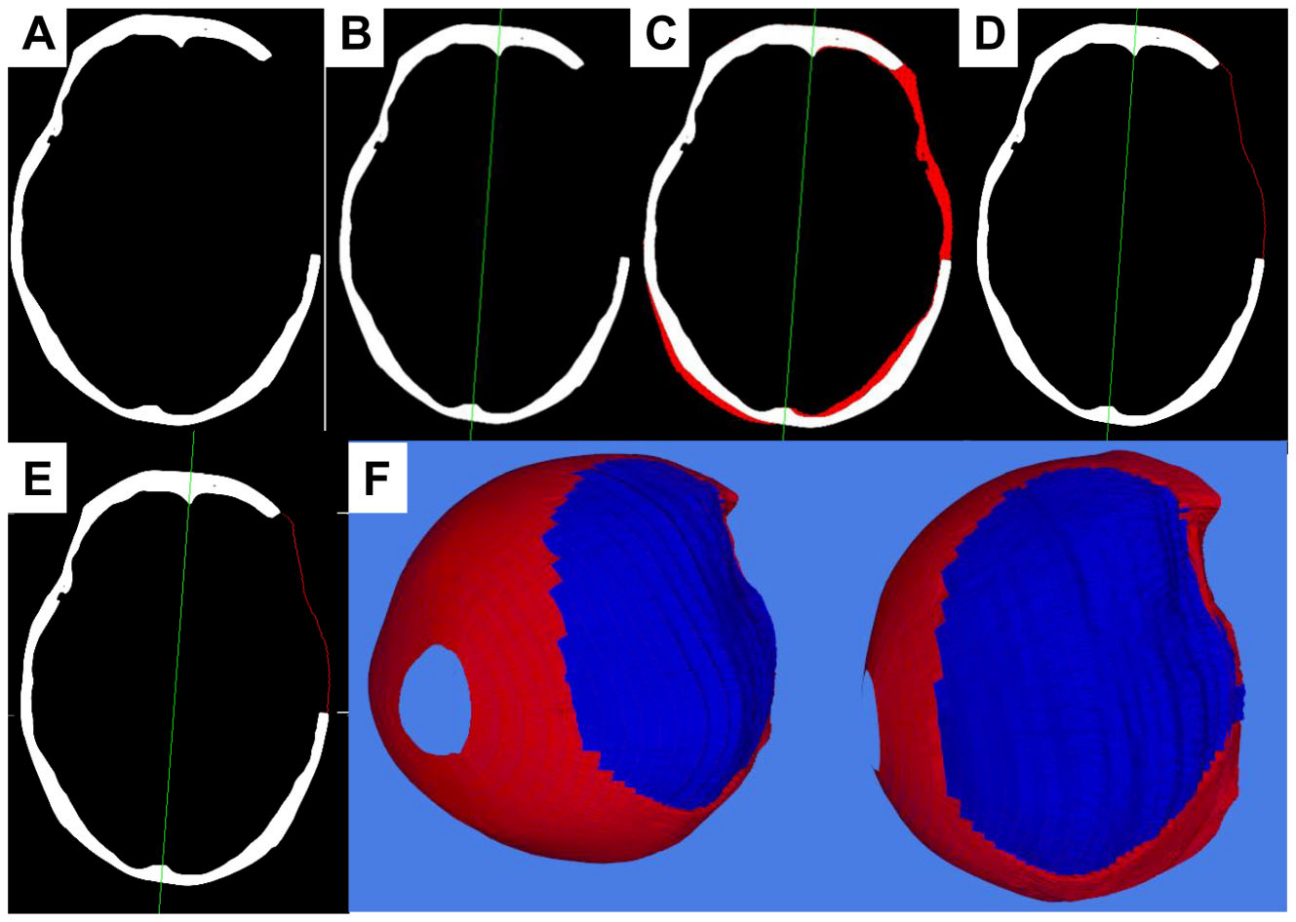
3-D CAD model for skull defect reconstruction of case 10 using OpenCV and OpenGL OBJ Viewer. (A). CT bone window image with left skull defect. (B). Axis of symmetry on CT bone window image. (C). Filled-image based on the axis of symmetry. (D). Reconstructed contour of skull defect. (E). Reconstructed contour with designated upper and lower boundaries (white line). (F). 3-D model showed by OpenGL OBJ Viewer. Reconstructed artificial flap is labeled in blue.

In the first step, OpenCV reads the 2-D images of brain CT scans in JPEG file format. CT bone window images with skull defects (Figure 1A) were used for reconstruction.

In the second step, the user should designate the axis of symmetry of skull in the CT bone window image according to the relative bony protrusions in the midline. The software allows the user to change the position of the axis of symmetry and show a preview of the axis with the CT bone window image. Figure 1B shows an example of the designated axis of symmetry (the green line) and the original CT bone window image.

The symmetric contour for the skull defect will be generated by OpenCV after the axis of symmetry is designated. In the third step, OpenCV fills the defect according to the contralateral counterpart based on the axis of symmetry. The filling parts are shown in red (Figure 1C). However, there are many redundant parts after this procedure.

In the fourth step, OpenCV only keeps the reconstructed contour of the skull defect and eliminates other red parts as shown in Figure 1D.

According to Figure 1D, we can still find some remnant, redundant contour at the top right corner of the image. This happens because the original skull is not fully symmetric. To correct this problem, in the fifth step, the user can designate the upper and lower boundaries of the reconstruction in this image as the gray bars shown in Figure 1E. By repeating step 2 to 5 for all slices of CT bone window images, OpenCV could generate all contours of the skull defect for all slices of the CT scans.

### Demonstrating 3-D models using OpenGL OBJ Viewer

To build the 3-D model of reconstruction of the skull defect, the software first extracts points surrounding the contour in each CT bone window images. Then, it connects the adjacent points to form triangles. Assuming there are *N_L_* surrounding points in image *L* and *N_L+1_* surrounding points in image *L+1*, the adjacent point in image *L+1* of the *m^th^* point in image *L* is the (m * N_L+1_/N_L_)^th^ point in image *L+1*. Let n = (m * N_L+1_/N_L_), for the *m^th^* point in image *L*, the software forms one triangle by connecting the *m^th^* and *(m+1)^th^* points in image *L* and the *n^th^* point in image *L+1*, and the other triangle by connecting the *(m+1)^th^* point in image *L* and the *n^th^* and *(n+1)^th^* points in image *L+1*. By forming two triangles for each point in each image, the software generates all triangles surrounding the surface of the skull. To discriminate the original bone from the newly reconstructed bone, the original bone is colored as red part and the newly reconstructed bone is colored as blue part. The generated 3-D model is presented in OBJ file format. The software utilizes OpenGL OBJ Viewer to show the 3-D model. Figure 1F shows an example of the 3-D model.

**Figure 2:**
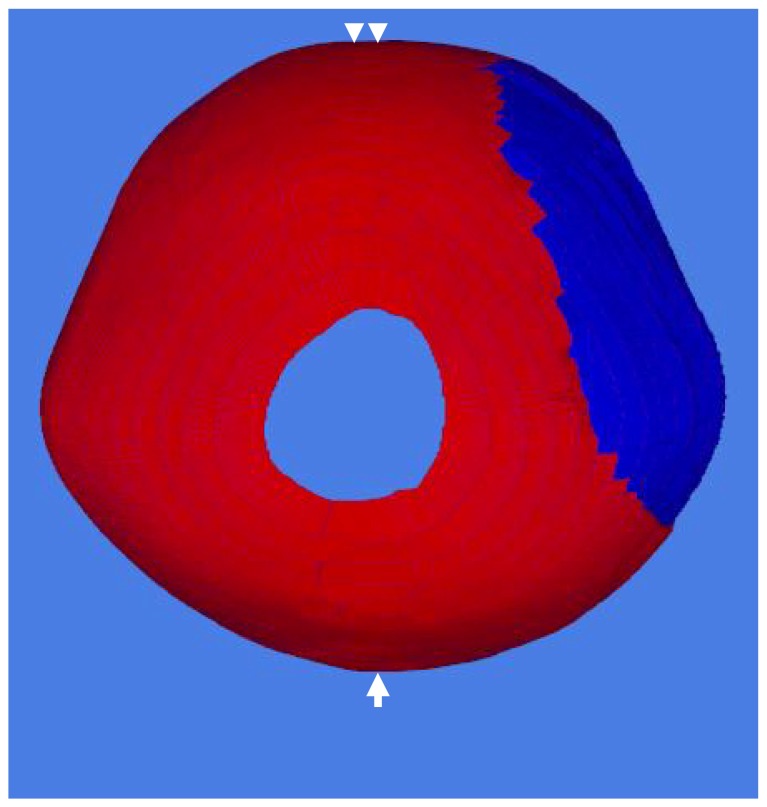
Verifying 3-D skull model reconstruction using CIS score of case 10. 3-D CT reconstructions were rotated as in a vertex view, with face up (double arrow) and in occipital inferior (single arrow) position. This corresponding vertex position of 3-D CAD model of the picture determined CIS in our patients. A digital camera took a picture of the 3-D CAD model from the vertex and the CIS score was determined.

In OpenGL OBJ Viewer, the user can rotate, zoom in and zoom out to change the viewpoint of the 3-D model. Thus, 3-D CT reconstructions can be positioned in a vertex view (Figure 2), which corresponds to the picture determining CIS in our patients.

### Symmetry measures of 3-D CAD reconstructions using CIS

CIS, described by Zonenshayn et al., is widely used in studying the 3-D craniofacial anatomy in plagiocephaly and brachycephaly [15]. We previously conducted the CIS method to measure aesthetic outcomes in cranioplasty with CAD/CAM titanium [4] and reformed polymethyl methacrylate (PMMA) [16] implants. The image of 3-D CT reconstructions in a vertex view was captured. Computer analysis of these images determines the shape of the 3-D reconstructions and the area of each hemisphere. A CIS value is calculated, with 100% signifying perfectly symmetric head. A score of 95% or more indicates symmetry [4], [14]–[16].

### Statistical analysis

Statistical analysis (Wilcoxon matched pairs signed rank test) was performed using the GraphPad prism software (GraphPad Software Inc., San Diego CA, USA). Significance was defined as p<0.05.

## Results

### Patient characteristics

From January 2011 to June 2012, 15 consecutive patients of major head trauma and stroke underwent DCs. There were 7 males and 8 females with a mean age of 66.3±18.2 years (range 34–90 years). Patient data are presented in Table 1.

**Table 1:** Patient demographic data and CIS scores.

						DC	
Patient	Age (years)	Sex	Cause for DC	TBI mechanism	Hematoma location	Uni+HR/Bil+HR	CIS of regular CAD models
1	57	F	TBI	Impact	Acute EDH, right T	Uni+HR	99.29
2	84	F	TBI	Motor vehicle accident	Acute SDH, right T	Uni+HR	99.24
3	86	F	Hemorrhagic stroke	-	ICH, left BG	Uni+HR	99.36
4	40	M	Hemorrhagic stroke	-	ICH, left BG	Uni+HR	99.12
5	34	F	TBI	Motor vehicle accident	Acute EDH,SDH, right F-T	Uni+HR	98.74
6	53	F	TBI	Fall	Acute EDH,SDH, left T-P	Uni+HR	98.47
7	90	F	TBI	Fall	Acute SDH, right F-T	Uni+HR	98.59
8	87	M	TBI	Fall	Acute SDH, left F-T	Uni+HR	99.26
9	60	M	Hemorrhagic stroke	-	ICH, right BG	Uni+HR	99.09
10	56	M	TBI	Impact	Acute SDH, left F-T; acute ICH, left F	Uni+HR	98.98
11	66	M	Hemorrhagic stroke	-	ICH, right BG	Uni+HR	99.34
12	75	M	TBI	Motor vehicle accident	Acute SDH, right T	Uni+HR	99.83
13	47	M	Hemorrhagic stroke	-	ICH, left BG	Uni+HR	99.71
14	78	F	TBI	Fall	Acute SDH, left F-T	Uni+HR	99.82
15	81	F	TBI	Fall	Acute EDH,SDH, ICH, right T-P	Uni+HR	99.63

M, male; F, female; TBI, traumatic brain injury; EDH, epidural hematoma; SDH, subdural hematoma; ICH, intracerebral hematoma; BG, basal ganglion; F, frontal; T, temporal; P, parietal; DC, decompressive craniectomy; Uni+HR, unilateral craniectomy+removal of hematoma; Bil+HR, bilateral craniectomy+removal of hematoma; CIS, cranial index of symmetry; CAD, computer-assisted design.

### CIS calculations of 3-D CAD reconstructions

We rotated the reconstructed 3-D CAD models to a vertex view with face up and occipital inferior position. A digital camera took a picture of these 3-D CAD models from vertex. CIS scores were normally distributed, with a mean score of 99.24±0.004% (range 98.47–99.84).

### Wilconxon matched-pairs sign rank test for CIS scores of 3-D CAD reconstructions

Since a CIS score of 95% or more indicates symmetry, we first examine the difference between 95% and those of reconstructed CAD models to determine if there is a statistically significant. The null hypothesis is that the two samples are identical populations. To test the hypothesis, we apply the Wilconxon matched-pairs sign rank test to compare the matched samples. For the paired test, we set the “paired” argument as true. As the p-value turns out to be 0.001, and is less than the 0.05 significance level, we reject the null hypothesis. Thus, at the 0.05 significance level, we conclude that CIS scores of reconstructed CAD models and CIS scores of 95% are non-identical populations. These data indicate the extent of symmetry is superior to CIS value of 95%.

A CIS value of 100% signifies perfectly symmetry. Assuming CIS scores of reconstructed CAD models and CIS value of 99.5% are identical populations and use the two-tailed Wilcoxon matched pairs signed-rank tests, we failed to reject our null hypothesis (p = 0.064). Hence, we postulate that CIS scores of reconstructed CAD models and CIS scores 99.5% are identical populations. CIS scores of CAD reconstructions are closely approaching 99.5%, indicating highly accurate symmetry. Moreover, CIS scores were statistically significantly lower than 99.6% (p = 0.021) in our study. Therefore, CIS scores of these CAD models were statistically significantly greater than 95%, identical to 99.5%, but lower than 99.6%.

### Clinical correlation

**Figure 3:**
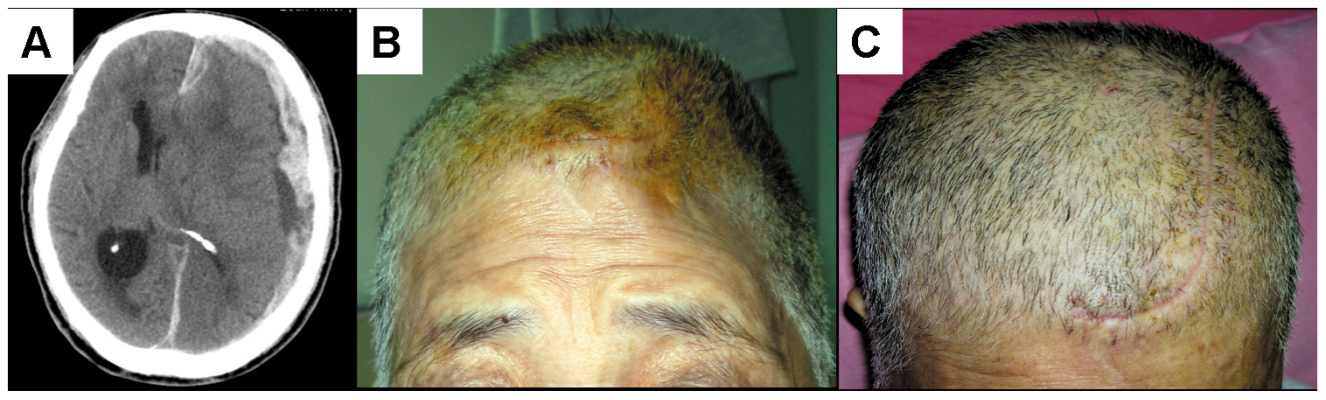
Representative photographs of case 14 before and after cranial implant reconstruction. (A). Initial CT scan of case 14, revealing acute subdural hemorrhage in the left temporal region with significant mass effect. (B). Patient's forehead appearance following DC due to TBI with acute subdural hematoma. (C). CAM-derived implant is based on regular 3-D CAD model in this case. The appearance of forehead following cranioplasty shows that reconstruction is symmetrical and stable after 20 months.

Since highly accurate CAD model enables manufacturing of highly symmetric CAM implant with superior cosmetic outcome, clinical photographs of pre- and post- cranial implant reconstruction fabricated from a regular CAD reconstruction are presented in Figure 3.

## Discussion

The present study introduces an assessment method using CIS analysis in 3-D CAD reconstructions of defective skull skeletons. The results of 15 CAD reconstructions in patients who underwent DCs were retrospectively reviewed. Our findings indicate that: 1) CIS calculation is useful to assess aesthetic outcomes of CAD-reconstructed skulls in terms of cranial symmetry; 2) CIS scores of these regular CAD models are statistically proven to be greater than 95% and identical to 99.5%, indicating highly accurate reconstruction. Our study provides a novel inspection protocol to show how 3-D CAD models are examined using CIS analysis. In clinical practice, smaller CAD/CAM implants are indicated in cranioplasty to prevent the impairment of scalp adaption. For this purpose, firstly we suggest examining regular CAD models using our method to verify the accuracy and symmetry. Secondly, based on the established CAD models, we can design CAD reconstructions including algorithms for depressed contours. Similarly, we can verify these models with depressed contours by CIS analysis to prevent the production of unsymmetrical CAD products.

To achieve a successful cranioplasty, tension-free skin closure, adequate vascularity of the scalp flap, preservation of soft tissue, and the integrity of dura are all necessary [4], [14]. There may be difficulties in scalp adaptation for cranioplasty in elderly patients, or in circumstances such as the ‘syndrome of the trephined’, which a sunken, flattened and thin skin flap may develop [16], [17]. However, difficult scalp adaptation consequently resulted in impaired tension-free skin closure. In addition, if a portion of the brain parenchyma has been removed during the initial decompressive surgery in the treatment of TBI, there will be a sub-cranioplasty dead space created [4]. In such situations of excessive wound tension and sub-cranioplasty dead space, the CAD technique should include algorithms to reconstruct the contour with an intended depression to prevent enlargement of the dead space between dura and implant. To avoid the production of unsymmetrical and unaesthetic CAM implants due to inadequate depression of CAD contours, the proposed aims of the current study is to establish amenable regular CAD models and quantitatively check these models by CIS analysis. Based on this verified regular 3-D CAD models, the surgeons can design modified CAD models including algorithms of depressed contours. Previously, we demonstrated aesthetically proven CAD/CAM titanium implants involving algorithms with a 1 to 2 mm contour depression to prevent excessive tension to the scalp and decrease sub-cranioplasty dead space. The ultimate cosmetic results of CIS scores in 40 patients with 49 3-D titanium mesh implants were 97.98±1.17% (range 96–99.8), indicating excellent cosmesis and craniofacial symmetry [4].

A growing body of literature addresses 3-D CAD models for skull reconstructions [12], [18]–[20]. A wide variety of commercial software is useful to reconstruct skull CAD models, including OpenCV, OpenGL OBJ Viewer, GLC Player, etc. Through CAD/CAM procedures, satisfactory CAM-derived alloplastic implants are made accordingly to the highly accurate 3-D CAD modeling. Therefore, CAD designs for 3-D reconstruction of skull skeletons are regarded as pivotal pre-processing procedures in the creation of CAM cranial implants. We suggest the use of our method to verify that: 1) whether any available 3D software that is designed to create a symmetric 3-D reproduction actually works; 2) whether it is acceptably symmetrical using the CIS measurement.

Previously, we reported that soft tissue handling contributes to craniofacial symmetry as importantly as does implant reconstruction in cranioplasty for skull defects. The more superficial soft tissue bulk we could preserve, the less soft tissue atrophy and the better maintenance of the outer appearance following cranioplasty [14]. Although the pivotal element of craniofacial symmetry is not solely restricted to implant reconstruction, we believe that highly accurate CAM-derived implant reconstruction plays a major role in the ultimate cosmetic result. Highly accurate CAD/CAM design with regular or depressed contour enables highly symmetric CAM-derived implant and therefore superior cosmetic outcome.

There are some limitations that should be considered. Although the verification method is simple and practical, the study included a relatively small number of cases and did not include CAD models with bilateral skull defects. Moreover, the optimal height of contour depression to achieve perfect aesthetic CAM implants has not been determined. Further reduced height-CIS relationship should be studied. The definitive decreased height to produce CAD models with CIS scores close to 95% should be obtained.

In conclusion, our data supported the highly accurate symmetry of these CAD models with regular contours in our study. We demonstrated the successful application of CIS analysis in the verification of 3-D CAD reconstructions with regular contours for the repair of cranial defects. The verification of regular 3-D CAD modeling is beneficial to symmetry analysis of CAD reconstructions with depressed contours in further work.
